# Selecting a specialized education database for literature reviews and evidence synthesis projects

**DOI:** 10.1017/rsm.2024.11

**Published:** 2025-04-01

**Authors:** Sarah Rose Fitzgerald, Kari D. Weaver, Alissa Droog

**Affiliations:** 1 University of Massachusetts, Amherst, MA, USA; 2 University of Waterloo, Waterloo, ON, Canada; 3 Northern Illinois University, DeKalb, IL, USA

**Keywords:** database coverage, database selection, education, education databases, evidence synthesis, systematic reviews

## Abstract

While the Institute of Education Science’s ERIC is often recommended for comprehensive literature searching in the field of education, there are several other specialized education databases to discover education literature. This study investigates journal coverage overlaps between four specialized education databases: Education Source (EBSCO), Education Database (ProQuest), ERIC (Institute of Education Sciences), and Educator’s Reference Complete (Gale). Out of a total of 4,695 unique journals analyzed, there are 2,831 journals uniquely covered by only one database, as well as many journals covered by only two or three databases. Findings show that evidence synthesis projects and literature reviews benefit from the careful selection of multiple specialized education databases and that ERIC is insufficient as the primary education database for comprehensive searching in the field.

## Highlights

**What is already known**

- As evidence synthesis projects in education inform policy, curriculum, and instructional practices, evidence synthesis teams should choose database(s) carefully to ensure that their review is comprehensive.

**What is new**

- This study compares the journal coverage overlap between four specialized education databases to recommend which should be used for evidence synthesis projects.
- This study contradicts current recommendations to use ERIC as the sole specialized education database for evidence synthesis searches.

**Potential impact for RSM readers**

- The findings of this study indicate that evidence synthesis projects in education should: select databases carefully; not rely on ERIC alone for a comprehensive literature search; and search more than one specialized education database.
- Contrary to existing guidelines, researchers should use more than ERIC (IES) as the core Education database and may wish to consult Education Source (EBSCO) in addition to ERIC for a more comprehensive literature search.

## 1 Introduction

Evidence synthesis projects like systematic and scoping reviews are growing in popularity in the field of education.^1^ Database selection is a critical aspect of evidence synthesis projects. A library database is a searchable electronic collection of published works such as journal and magazine articles. Library databases are often sold via subscription to libraries by vendors that index and compile publications from a variety of titles. Many vendors sell dozens or even hundreds of different databases with different topical foci or different coverage. Since the sample of studies used in an evidence synthesis project determines the quality of the review, it is important to select the most suitable databases.^2^ Within the field of education, the current recommendations for subject-specific database selection have not been evaluated in many years and warrant examination, particularly as the use of evidence synthesis methods becomes increasingly widespread.

The authors of this study regularly notice that many education researchers do not have a thorough grasp of the scope and limitations of the specialized databases they use for evidence synthesis. Some scholars mistake a vendor who provides multiple databases like EBSCO or ProQuest with an individual database provided by that vendor like Academic Search Complete (EBSCO). Conflating the database name with the vendor is so common in education that the most popular research methods textbook in the field makes this mistake (p. 29).^3^ This obscures the methods of a paper and prevents reproducibility. An accurate explanation of the choice of database or databases is impossible without accuracy in identifying the database used. Additionally, common resources for writing scholarly articles advise searching multiple databases but do not give field-specific advice.^4^ There is an expectation that scholars will use subject-specific databases in combination^3^ with multidisciplinary databases for comprehensive coverage.^5^ ^–^ ^9^ Since many libraries do not have access to all the specialized databases needed to conduct comprehensive searches, popular texts for learning to write research papers advise scholars to investigate libraries beyond their own.^10^ Therefore, the selection of databases is a problem that needs attention and clear, evidence-based recommendations in the field of education. This study seeks to fill this gap in understanding by evaluating the coverage overlaps and discrepancies between the premier specialized education databases from four vendors.

### 1.1 Popularity of the ERIC database in education

Within the field of education, the most popular subject-specific database is the Educational Resources Information Center (hereafter ERIC).^6^ ^,^ ^11^ ^,^ ^12^ ERIC is provided by the Institute for Education Sciences (hereafter IES) and funded as part of the United States Federal Department of Education. In addition to the free version from IES, ERIC can also be searched from a number of vendor-supplied platforms including ProQuest, EBSCO, OVID, and FirstSearch. Although few guidelines exist for selecting databases in the field of education, current recommendations for evidence synthesis projects recommend ERIC. The What Works Clearinghouse creates respected evidence-based recommendations for the field of education, and its *Procedures and Standards Handbook* recommends ERIC as “the primary database” for literature and systematic searches (p. 145).^6^ In addition, the recent book, *Systematic Reviews in Educational Research: Methodology, Perspectives, and Application*, recommends searching a variety of sources, but names only ERIC for subject-specific databases in education (p. 9).^11^ Consequently, researchers are left without evidence-based direction on the selection of subject-specific databases for evidence synthesis in education beyond ERIC.

### 1.2 Database comparison methods

Database comparison studies enable researchers to make informed decisions regarding database selection and generally employ one of four methods: keyword-level, core-list, ISSN-level, and other methods. Keyword-level database comparisons allow researchers to evaluate which database covers a topic best. They do this by comparing search results between databases using the same keywords.^13^ ^–^ ^17^ Core-list database comparisons evaluate which databases index a set of pre-defined core journals in a field.^12^ ^,^ ^18^ Unlike core-list comparisons, which are based on some ideal core list of journals for a field, ISSN-level database comparisons directly compare the ISSNs included in two or more databases. ISSN-level database comparisons seek to understand coverage overlaps between databases using vendor-supplied journal titles or ISSN lists.^19^ ^–^ ^22^ Finally, there are emerging methods for database comparisons which evaluate the size of databases,^23^ identify funding agencies,^15^ compare citation counts,^24^ ^,^ ^25^ or compare search functionality.^2^ Many tools are now available to libraries that can compare databases at the ISSN level.^26^ However, the comparisons resulting from these tools often have discrepancies,^21^ and are rarely shared beyond the library. This leaves few recommendations for researchers about coverage overlap between databases to inform evidence synthesis. As evidence synthesis projects strive for a comprehensive search, the ISSN-level comparison is most applicable to identifying coverage comparisons that could inform this work in education.

### 1.3 Previous studies have not favored ERIC

Although dated, previous database comparisons which included ERIC have called into question its ubiquitous use. Using a keyword-level database comparison in 2000, Black et al.^27^ found that researchers should search ERIC among other specialized and non-education databases for comprehensive coverage of a topic. After ERIC underwent a major change between 2003 and 2004, a few publications reviewed its coverage and compared it to other databases. Strayer^12^ used an ISSN-level database comparison to review the old and new versions of ERIC with a variety of other databases, finding that ERIC should be used in conjunction with other databases such as Education Research Complete (EBSCO). Corby^28^ also conducted a variation of an ISSN-level comparison of specialized education databases and also found that Education Research Complete (EBSCO) indexed the most journals out of the specialized education databases included in the study. Finch^29^ conducted a keyword-level database comparison on the topic of gifted education, finding that ERIC had more unique articles on the topic selected in comparison to Education Full Text (now Education Source [EBSCO]).

ERIC has several limitations which impact its utility for some evidence synthesis projects. First, ERIC’s collection policy prioritizes “content including participants in the United States” (p. 2).^30^ Since ERIC is funded by the United States Federal Department of Education, this is unsurprising but does limit its coverage for international education research. Second, ERIC also selectively indexes many of the journals it includes^30^ ^,^ ^31^ and does not share publicly which journals are selectively or comprehensively indexed. Although this study is concerned with coverage rather than functionality, a third challenge with using ERIC is choosing which platform to search. Concerns about the ERIC public interface via the IES platform (https://eric.ed.gov) have been raised in relation to evidence synthesis methods (pp. 208, 197).^2^ While the performance limitations of the IES platform may be mitigated by using ERIC through vendor-supplied interfaces, specifically EBSCO,^2^ these options contain a documented time lag for the inclusion of new content (p. 145).^6^ The collective issues raised by past analyses of ERIC, the limitations in indexing and international coverage, and the concerns over functionality based on the platform further justify the need to objectively compare the coverage of specialized education databases rather than simply defaulting to ERIC as a standalone recommendation.

### 1.4 Need for an updated education database comparison

Despite the varied and continuing concerns around the use of ERIC as the primary resource for literature in education, its use as the solitary specialized education database in evidence synthesis persists. As database selection impacts the quality and rigor of evidence synthesis projects, and database coverage changes regularly, it is necessary to have updated guidance on the coverage and recommended selection of databases in education to inform these growing methods. Scholars in the field of education also have a particular responsibility to understand and make informed and intentional decisions about where to gather evidence as their work subsequently informs curricular, instructional practice, and policy decisions. The distance between the recommendations for database selection and the common practices in the field of education necessitates an in-depth look at specialized education database coverage within the field. This ISSN-level database comparison study addresses this gap by examining the coverage overlaps between four specialized education databases to provide updated guidance for the selection of subject databases in education evidence synthesis projects.

Research questions:

1. What are the journal coverage overlaps of specialized education databases?
2. What are the implications of these journal coverage overlaps for selecting specialized education databases as part of research in education?

## 2 Methodology

### 2.1 Database selection

To investigate the coverage overlaps and unique contributions of specialized general education databases, we first considered which databases to analyze. We considered 13 specialized education databases and selected four of the largest specialized general education databases from four vendors for this analysis (see the Appendix). We chose not to include interdisciplinary databases in order to focus our examination on subject-specific databases and inform evidence-based selection of these tools as a component of evidence synthesis projects in the field of education. We considered databases from vendors including the IES, ProQuest, EBSCO, and Gale. We also reviewed library research guides at 10 top graduate schools for education^32^ to see which databases were recommended for education research. For our analysis, we selected Education Source (EBSCO), Education Database (ProQuest), ERIC (IES), and Educator’s Reference (Gale). EBSCO has many specialized education database products, so we picked Education Source, their largest database (3,537 journals) as their other databases are subsets of the titles contained by Education Source.

### 2.2 Challenges with material type selection and indexing

Once we selected our databases for analysis, we procured title lists for each database (in December 2022). Title lists were easy to obtain from ProQuest, Gale, and EBSCO, but required emailing the IES several times to obtain the title and ISSN/E-ISSN lists required for the analysis.

One challenge with vendor-supplied data is the opacity around the selection procedures for the inclusion of a publication in the database products. Of the databases considered, only ERIC (IES) publishes their selection and indexing procedures, with the other major vendors treating those procedures as proprietary. ERIC (IES) describes their procedure as selecting publications with 80% or more education-related content that is assessed by a review of, “… three years of current journal issues during a review of a source” (p. 7).^30^ Our analysis includes all journals from the database title lists, including those that are currently published and included, as well as journals that are no longer in publication, journals with selective coverage, or lapsed coverage in which older issues are included, but not current issues. We did not limit our analysis to currently indexed journals because scholars engaged in literature synthesis projects are often interested in how a topic has changed over time, meaning older publications are relevant to their research. Indeed, there is no way for a searcher to exclude literature which is no longer indexed when searching these databases. That said, IES was only able to provide a list of journals that are currently indexed (selectively or fully) and was unable to supply the titles of journals with lapsed indexing. We acknowledge that the list of ERIC titles we used for this analysis does not reflect the full list of titles available through ERIC because IES does not track that full list. Therefore, we also repeated our analysis with only currently indexed journals for all databases to ensure that the findings of this study were not being skewed by ERIC’s exclusion of lapsed coverage journals.

Another challenge working with vendor-supplied data is that each of the vendors categorizes the indexed journals differently (Table 1). In the applied field of education, it is the convention to incorporate scholarly and trade (often called practitioner) journals in literature reviews (pp. 71–73).^33^ Therefore, practitioner/trade journals and magazines serve an important function and are often included in evidence synthesis projects in education. For this reason, we limited our analysis to the most consistent categories which reflected scholarly and practitioner journals, excluding other categories of publications such as blogs, books, conference proceedings, images, reports, government documents, newsletters, newspapers, videos, and working papers.

**Table 1:** Journal categories by database

Database	How journals are categorized by database	Journals (#)
Education Source (EBSCO)	Magazine	808
	Academic journal	2,663
	Trade publication	66
Educator’s Reference Complete (Gale)	Magazine/journal	1,221
Education Database (ProQuest)	Scholarly journal	1,315
	Trade journal	126
	Magazine	120
ERIC (IES)	Journal	1,304

### 2.3 ISSN-level coverage comparison

Our first step to compare the databases in our sample was to match ISSN or E-ISSN numbers where they were available. This was challenging because some titles had only an ISSN, others had only an E-ISSN, and others had both. We had to ensure that we did not count titles with multiple identifiers more than once. ISSN/E-ISSNs were matched in Excel using the VLOOKUP function for every possible combination of overlaps between the databases. This included running 21 VLOOKUP formulas across the title lists. After this ISSN analysis was completed, we manually compared 460 titles for which no ISSN or E-ISSN numbers were available (Table 2). This was completed by having a member of the research team investigate potential matches, and a second member of the research team verify those matches. The team completed a third pass of six potential title matches that were the most challenging to compare due to different naming conventions.

**Table 2:** Titles in each database with/without ISSNs

	Education source	Education database	Educator’s reference	ERIC
	(EBSCO)	(ProQuest)	(Gale)	(IES)
Titles with ISSN/E-ISSNs	3,150	1,529	1,195	1,291
Titles without ISSN/E-ISSNs	387	33	26	14
Total titles analyzed	3,537	1,562	1,221	1,305

## 3 Results

**Figure 1:**
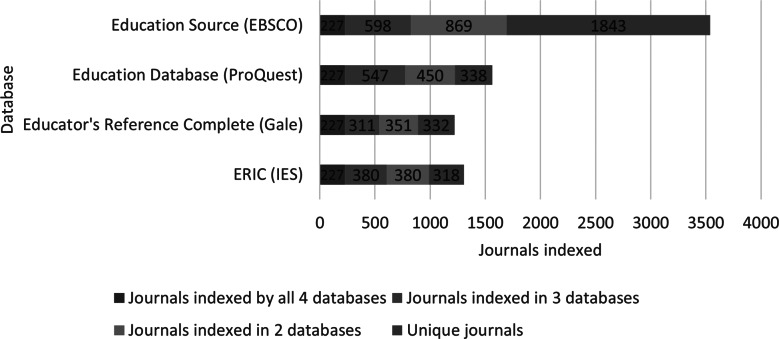
All indexed shared journals between education databases.

Figure 1 shows that the four databases investigated have few titles in common and many titles which are unique to only one database. Each database offers at least a few hundred titles which none of the others offer. Education Source (EBSCO) contains the most unique titles in education with 1,843. The number of titles indexed by all four databases is only 227. The sum of Figure 1 is 7,625.

Table 3 displays the number of journals shared across all four databases, shared between the various subsets of databases, and the number of journals unique to a single database. ERIC is the least unique of the databases. ERIC overlaps with Education Source (EBSCO) on 860 journals which is 66% of its total coverage (1,305 journals).

**Table 3:** Journal overlaps between specialized education databases

Database(s)	Journals indexed
Journals that are indexed in four databases (227)	
Education Source (EBSCO), ERIC (IES), Education Database (ProQuest), and Educator’s Reference Complete (Gale)	227
Journals that are indexed in three databases (612)	
Education Source (EBSCO), ERIC (IES), and Education Database (ProQuest)	301
Education Source (EBSCO), Educator’s Reference Complete (Gale), and Education Database (ProQuest)	232
Education Source (EBSCO), ERIC (IES), and Educator’s Reference Complete (Gale)	65
ERIC (IES), Educator’s Reference Complete (Gale), and Education Database (ProQuest)	14
Journals that are indexed in two databases (1,025)	
Education Source (EBSCO) and Education Database (ProQuest)	325
Education Source (EBSCO) and Educator’s Reference Complete (Gale)	277
Education Source (EBSCO) and ERIC (IES)	267
ERIC (IES) and Education Database (ProQuest)	82
Educator’s Reference Complete (Gale) and Education Database (ProQuest)	43
ERIC (IES) and Educator’s Reference Complete)	31
Journals that are unique to one database (2,831)	
Education Source (EBSCO)	1843
Education Database (ProQuest)	338
Educator’s Reference Complete (Gale)	332
ERIC (IES)	318

**Figure 2:**
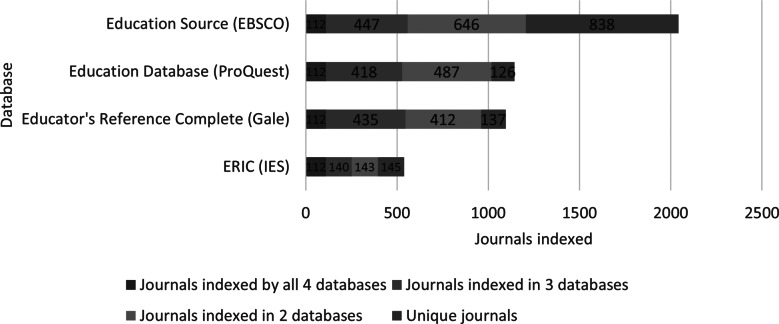
Currently indexed shared journals between education databases.

Figure 2 shows the same analysis of journals from the four databases including only actively published titles. The same pattern is visible in which there are many titles unique to a single database and many more which are shared by only two or three of the databases. Again, ERIC is the least unique and EBSCO’s Education Source is the most unique.

Table 4 displays the differences in the total number of titles contained in the four databases of study versus the number of actively published titles.

**Table 4:** Comparison of all indexed journal titles versus just currently indexed titles

	Education source	Education database	Educator’s reference	ERIC
	(EBSCO)	(ProQuest)	complete (Gale)	(IES)
Currently indexed journals	2,073	1,098	540	1,131
All indexed journals	3,537	1,562	1,221	1,305

## 4 Discussion

### 4.1 Choose database(s) carefully and justify the choice

Rather than relying on ERIC without investigating or explaining the choices, education researchers should make informed choices for the database(s) they use and describe their decision-making process. This study found that none of the specialized education databases comprehensively covers journals in the field. This reinforces for the field of education that researchers engaged in evidence synthesis should explicitly name the database(s) and vendor platform(s) used for the search.^8^ The choice of databases should be accompanied by a justification for the decision which should be informed by the coverage of the databases selected. This approach would enhance the reliability of evidence synthesis projects in education. It is important to ensure quality in evidence synthesis projects which determine policy changes, teaching practice, intervention effectiveness, and posit theory.^34^ ^,^ ^35^

Many academic libraries provide a discovery search tool which can search content from multiple databases at a time. This can lead researchers to assume that such a tool searches every possible database. However, these discovery tools search only records from databases subscribed to by the library they serve and, depending on the library, may only show results for records with full text. A given library might subscribe to one or many education literature databases. To conduct a comprehensive literature search in education, scholars must investigate how many and which specialized education databases are available from their institution.

### 4.2 Do not rely on ERIC as your only education database

Current recommendations for evidence synthesis methods in education prioritize the use of ERIC as the main database for searching.^6^ ^,^ ^11^ This study reveals that ERIC (IES) had the least number of unique journals out of four specialized education databases. The findings of this study and older studies,^12^ ^,^ ^27^ ^,^ ^28^ indicate that focusing on ERIC as the primary education database is insufficient for education research. While some of these limitations emanate from the title coverage itself, some are also results of the way in which ERIC indexes publications.^31^ One must contact IES to obtain a full title listing for ERIC with ISSN data, title lists are limited to currently indexed titles, and it is unclear how many titles are lapsed or selectively indexed.^30^ The ERIC database is available from multiple vendors, and it is also important to select the platform with intention. There is a time lag between articles being added to the IES version and other vendor-supplied platforms for ERIC. However, there is also evidence that the EBSCO platform has better functionality for evidence synthesis projects than the IES version.^2^ Researchers are likely to continue using ERIC for its strong coverage of educational reports and gray literature but should pair it with at least one other specialized education database for evidence synthesis projects.

### 4.3 Use more than one specialized education database

Based on the overlap analysis conducted in this study, education researchers need to use more than one specialized education database. If there is a reason a researcher or team must limit their search to a single education database, then Education Source (EBSCO) stands out as the database with the most titles. Even institutions with strong education programs may make only one or two education databases available through their academic libraries. This study indicates scholars working on evidence synthesis projects in education would be well advised to take advantage of public use policies at other libraries and collaborate across institutions to access all the databases needed for comprehensive work. Due to the presence of selective indexing and abstract-only databases in education, evidence synthesis projects are also likely to require extensive use of interlibrary loan for full-text access. Multidisciplinary databases are also important to support comprehensive literature searches. Since selecting among specialized databases requires expertise on how databases work and what they contain, librarians should be consulted on the selection of databases for literature reviews and evidence synthesis. Including librarians in the selection of databases would support recommendations from this study to carefully choose databases and explain those choices while bringing rigor and consistency to evidence synthesis projects.^7^ ^,^ ^8^ ^,^ ^36^ ^,^ ^37^

## 5 Limitations

Working with vendor-supplied data introduced some limitations to our study. Our analysis is only as good as the data we input. The title counts that underpin this analysis are a snapshot of a particular moment in time (December 2022) since database vendors move titles in and out of their databases.

- We did not verify that every title covered by the databases we examined was education-related. Since that was beyond the scope of this study, we trusted that the database vendors included mostly education-related journals in their education database products. While ERIC is focused on education, it does have journals related to other social science fields like Psychology, Arts and Humanities, and Medicine (p. 2724).^14^
- The various vendors whose products we examined categorize titles differently. For example, EBSCO and ProQuest make distinctions between academic journals, magazines, and trade journals, while Gale and ERIC group all three of these publication types into a single category, so we could not limit our investigation to only academic journals.
- Vendors do not always index every item from every journal they include in their title lists. For example, ERIC selectively indexes articles on the topic of education for some journals (p. 7).^30^
- The vendor-supplied title list from ERIC (IES) excludes any journals that are no longer indexed in the database, which will make ERIC’s size appear smaller than it is. The other database title lists include journals with lapsed coverage.
- Merely looking at the title count in each of the education databases does not give one a full picture of their relative coverage. One also must know how many of the total titles in each database are shared in multiple databases to understand how much unique content each database provides. Our analysis shows the number of titles which overlap across multiple education databases.

## 6 Future research

This study indicates there are limitations in specialized database coverage in the field of education which negatively impact the execution of evidence synthesis methods in the discipline. In addition to the potential relevance of this study’s method and findings to other disciplines, future study could introduce a greater level of granularity in the overlap analysis by looking at journal impact factors to determine coverage of highly cited journals across database products. Additionally, future work should aim to understand how database coverage of major subtopic areas in education differs. Perhaps one database is better than another for a topic like special education, but that is not known at present. Future research can also examine the functionality of the various specialized databases in education.

Future work should examine the impact of multidisciplinary databases in the field of education, including Academic Search Complete (EBSCO), the Web of Science, and Scopus, which contain wide-ranging subject coverage in education or other fields. Multidisciplinary databases can have value to education researchers as education is a field which draws on methods from a variety of disciplines. This work may be particularly important for scholars at less well-resourced institutions who may primarily need to rely on open-access resources or may only have one core education database and need to supplement their findings by using multidisciplinary databases or open-access materials. These steps would better guide scholars wishing to use evidence synthesis methods in the field of education and ensure due diligence for policy decisions emanating from evidence synthesis findings.

For researchers engaged in database comparison work, it is important to consider that this work is not straightforward. A title count offered by a vendor is likely to have several issues which are not visible at first glance. Vendors offer different variables in their title lists. Vendors choose different types of materials to include and exclude from their lists. Vendors categorize material types differently. Vendors include duplicates of ISSNs for a variety of reasons including change in title, change in publisher, or ambiguous material type. These duplicates must be removed to obtain an accurate title count. ISSNs are not really a unique identifier since some journals do not have ISSNs and many have both an ISSN and an eISSN which are used interchangeably by vendors. Comparisons by proprietary tools are risky to use for decision-making because the methods they use are not published and it is not possible to ensure that they are eliminating false or duplicative matches.

## 7 Conclusion

This analysis revealed that comprehensive education literature searching requires access to publications provided by multiple specialized education databases. As evidence synthesis projects inform policy, curricular, and instructional decisions, evidence synthesis teams should choose databases carefully and search in more than one specialized education database to ensure appropriate coverage. Education Source (EBSCO) was identified as the database with the widest coverage and should be searched if possible. This contrasts with the existing recommendations^6^ ^,^ ^11^ which emphasize using ERIC. Many academic library budgets do not permit the purchase of multiple education databases. One way to address this tension is by expanding the open access availability of education research. Another is for scholars to intentionally work in cross-institutional teams to enhance access to a wider range of specialized database options. Despite several limitations (see Limitations), this study makes a valuable contribution. The scale of the coverage overlaps between specialized education databases (or lack thereof) are significant enough that our three major recommendations (stated next) stand regardless of discrepancies caused by indexing limitations introduced by vendor-supplied data. Therefore, researchers in education should (1) choose database(s) carefully and justify the choice, (2) not rely on ERIC as your only education database, and (3) use more than one specialized education database.

## Data Availability

The data that support the findings of this study are openly available to download from database vendor websites and by request from the Institute of Education Sciences. The exact data that support the findings of this study are available from the corresponding author upon reasonable request.

## Appendix. Education databases considered for this study

**Table:**

	Journals indexed		
	(rounded to		
Database (vendor)	nearest 10)	Inclusion rationale	Exclusion rationale
ERIC (IES)	1,310	Most popular database in education	
Education Full Text (EBSCO)	560		Subset of Education Source (EBSCO)
Education Research Complete (EBSCO)	1,530		Subset of Education Source (EBSCO)
Education Abstracts (EBSCO)	780		Subset of Education Source (EBSCO)
Education Administration Abstracts (EBSCO)	210		Subset of Education Source (EBSCO), Specialized focus, not a general education database
Education Source (EBSCO)	2,210	Largest education database from EBSCO, contains journals from Education Full Text and Education Research Complete	
British Education Index (EBSCO)	445		Specialized focus, not a general education database
Professional Development Collection (ProQuest)	760		Specialized focus, not a general education database; small selection of journals
Education Database (ProQuest)	1,000	Largest education database from ProQuest	
Education Collection (ProQuest)	Unknown		Described as ERIC (IES) plus the Education Database (ProQuest)
Professional Education (ProQuest)	290		Too small for comparison
In context: For Educator’s (Gale)	Unknown		Specialized focus on lesson plans and instructional tools, not a general education database
Educator’s Reference Complete (Gale)	1,100	Largest education database from Gale	
